# Comparison of the mismatch-specific endonuclease method and denaturing high-performance liquid chromatography for the identification of *HBB *gene mutations

**DOI:** 10.1186/1472-6750-8-62

**Published:** 2008-08-12

**Authors:** Chia-Cheng Hung, Yi-Ning Su, Chia-Yun Lin, Yin-Fei Chang, Chien-Hui Chang, Wen-Fang Cheng, Chi-An Chen, Chien-Nan Lee, Win-Li Lin

**Affiliations:** 1 Institute of Biomedical Engineering, College of Medicine and College of Engineering, National Taiwan University, Taipei, Taiwan; 2 Graduate Institute of Clinical Medicine, College of Medicine, National Taiwan University, Taipei, Taiwan; 3 Department of Medical Genetics, National Taiwan University Hospital, Taipei, Taiwan; 4 Institute of Molecular Medicine, College of Medicine, National Taiwan University, Taipei, Taiwan; 5 Department of Obstetrics and Gynecology, National Taiwan University Hospital, Taipei, Taiwan

## Abstract

**Background:**

Beta-thalassemia is a common autosomal recessive hereditary disease in the Meditertanean, Asia and African areas. Over 600 mutations have been described in the beta-globin (*HBB*), of which more than 200 are associated with a beta-thalassemia phenotype.

**Results:**

We used two highly-specific mutation screening methods, mismatch-specific endonuclease and denaturing high-performance liquid chromatography, to identify mutations in the *HBB *gene. The sensitivity and specificity of these two methods were compared. We successfully distinguished mutations in the *HBB *gene by the mismatch-specific endonuclease method without need for further assay. This technique had 100% sensitivity and specificity for the study sample.

**Conclusion:**

Compared to the DHPLC approach, the mismatch-specific endonuclease method allows mutational screening of a large number of samples because of its speed, sensitivity and adaptability to semi-automated systems. These findings demonstrate the feasibility of using the mismatch-specific endonuclease method as a tool for mutation screening.

## Background

Beta-thalassemia is one of the most common genetic diseases in the world. It is an autosomal recessive inherited disease resulting from point mutations, small insertions, or deletions in the beta-globin (*HBB*) gene [1]. Over 600 mutations have been described in the HBB gene, of which more than 200 are associated with a beta-thalassemia phenotype [2,3]. The wide diversity of mutations in the *HBB *genes makes mutation screening time-consuming and expensive. In the Southeast Asian population, the common beta-thalassemia mutations include c.-78 A>G, c.2 T>A, c.52 A>T, c.84_85 insC, c.125_128 delTCTT, c.130 G>T, c.216_217 insA, and c.316-197 C>T [4].

A wide variety of methods for genotyping the *HBB *gene have been developed using different detection techniques based on different principles [5], including allele-specific oligonucleotide hybridization (ASO) [6], amplification refractory mutation system (ARMS) [7], allele-specific PCR [8], reverse dot blot [9], restriction fragment length polymorphism (RFLP) [10], single base extension (SBE) [11,12], and others [13-17]. These methods, however, are limited to the study of hotspot or known mutations. Additionally, single-strand conformation polymorphism (SSCP) [18,19], denaturing gradient gel electrophoresis (DGGE) [20], temporal temperature gel electrophoresis (TTGE) [21], are efficient techniques to screen for unknown mutations. The advantage of these methods above is that it is inexpensive and suitable for large-scale research applications. However, its major disadvantage is lower detection rates. Recently, chemical cleavage of mismatch (CCM) [22] analysis has been reported to be a promising tool for mutation detection with advantages of accuracy, simplicity, and cost effectiveness, but it involves the use of toxic chemicals.

Enzymatic mismatch cleavage methods have been around for some time and include the use of T4 endonuclease VII [23], endonuclease V (Endo V) [24], mung bean nuclease [25], S1 nuclease [26], and CEL I nuclease [27-29]. There are growing numbers of publications describing the use of mismatch-specific endonuclease in the mutation discovery methods known as Tilling and Ecotilling [30-32].

In this study, we used the mismatch-specific endonuclease method to detect disease-causing mutations in the *HBB *gene. The method was modified by amplifying the PCR products using three primers in the *HBB *gene labeled at 5' ends with fluorescent dyes. The results obtained using the mismatch-specific endonuclease method were compared with findings using denaturing high-performance liquid chromatography (DHPLC).

## Results

### Detection of HBB Mutations by the Mismatch-specific Endonuclease Method

**Figure 1:**
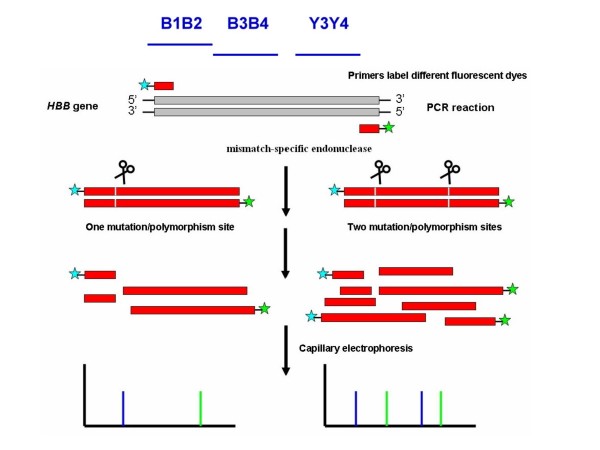
Principles of the mismatch-specific endonuclease cleavage of the heteroduplex formation using primers labeled with two different fluorescent dyes.

Chemical cleavage of mismatch (CCM) is a highly specific and sensitive method to induce mismatch-cleavages [33]. However, the major disadvantage of CCM is the use of toxic chemicals during the process in the reactions or buffers, which could be avoided by using enzymatic mismatch cleavage. The celery enzyme is a member of a family of plant endonucleases that recognizes mismatches in heteroduplex DNA and cleaves both strands on the 3' side of the mismatch distortion [27]. To investigate the diagnostic value of this approach, the exons of *HBB *genes were amplified by PCR using primers labeled at the 5' end with fluorescent dyes (Figure 1). We also employed a simple method to detect mutations in *HBB *genes using the mismatch-specific endonuclease cleavage and separation of the PCR products by capillary electrophoresis. The approach is based on mismatch-specific selective reactions of mismatched DNA cleavage by endonuclease and separation of the PCR product fragments by capillary electrophoresis. This method is also adaptable to automated capillary electrophoresis which could increase its speed, sensitivity, and reproducibility, making it suitable for large-scale and high-throughput mutation screening.

**Figure 2:**
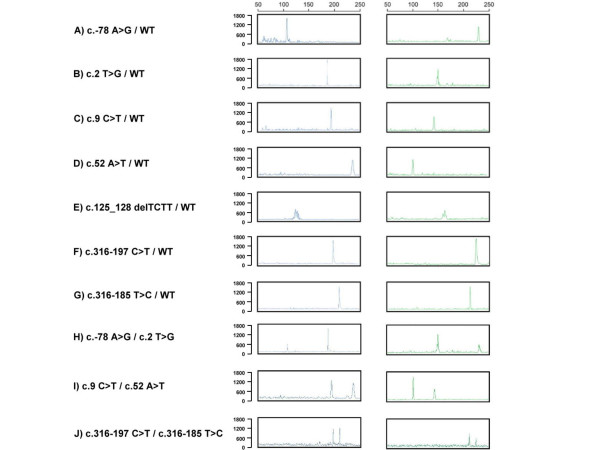
**Capillary electrophoresis analysis of *HBB *genes for PCR products cleaved by mismatch-specific endonuclease from*****A) **an individual with a genotype of c.-78 A>G/WT*, **B)** an individual with a genotype of c.2 T>G/WT, **C)** an individual with a genotype of c.9 C>T/WT, **D)** an individual with a genotype of c.52 A>T/WT, **E)** an individual with a genotype of c.125_128 delTCTT/WT, **F)** an individual with a genotype of c.316-197 C>T/WT, **G)** an individual with a genotype of c.316-185 T>C/WT, **H)** an individual with a genotype of compound heterozygous mutation c.-78 A>G/c.2 T>G, **I)** an individual with a genotype of c.9 C>T/c.52 A>T, and **J)** an individual with a genotype of c.316-197 C>T/c.316-185 T>C.

We detected all the DNA variants of the *HBB *gene including single base substitutions and deletions of TCTT. The different mutation sites and the expected sizes of PCR products cleaved by mismatch-specific endonuclease are described in Table 1. Samples of variant DNA fragments obtained with different base pairs were analyzed using capillary electrophoresis and the results are shown in Figure 2.

**Table 1:** Variation sites and the expected lengths of mismatch-specific endonuclease cleavage fragments for the human *HBB *gene

***Variations Sites***	***Nucleotide Change***	***Amplicon***	***Mutation Type***	***Expected Fragment Lengths (bp)***	
				***Observation with FAM labeling***	***Observation with HEX labeling***
c.-78	A>G	B1B2 (337 bp)	Regulatory	107	230
c.2	T>G	B1B2 (337 bp)	Misense	186	151
c.9	C>T	B1B2 (337 bp)	SNP	193	144
c.52	A>T	B1B2 (337 bp)	Nonsense	236	101
c.125_128	delTCTT	B3B4 (287 bp)	Frame shift	125	162
c.316-197	C>T	Y3Y4 (423 bp)	Splicing	198	225
c.316-185	T>C	Y3Y4 (423 bp)	SNP	210	213

The PCR fragment amplified by the B1 and B2 primers was 337 bp; this fragment was cut into fragments of 107 bp and 230 bp by mismatch-specific endonuclease indicatingthe presence of c.-78 A>G mutation (Fig. 2A). Similar to the c.-78 A>G variation, the common c.9 C>T single nucleotide polymorphism (SNP) of the PCR fragment was cleaved to 193 bp and 144 bp (Fig. 2C).

**Figure 3:**
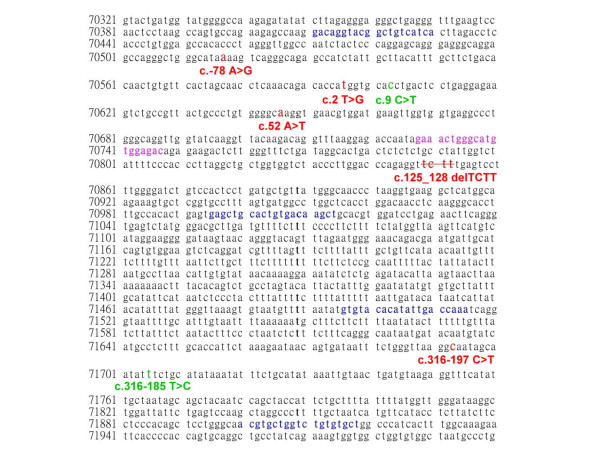
**Human beta-globin gene sequence (NG_000007), primer locations and the variation sites.** The primers are labeled in boldface letters and blue font, the mutation sites are labeled in boldface letters and red font, and the polymorphism are marked in boldface letters and green font. The overlap sequence is labeled in pink font.

The FAM- and HEX-labeled probes had the same sensitivity and specificity and could be used for double confirmation of the results. The formation of new cleavage products indicated the presence of variation, while their size provided some information about their location in the entire gene (Figure 3). The two variations could be also determined by the mismatch-specific endonuclease method as shown in Figure 2. The 107 bp, 230 bp, 186 bp, and 151 bp cutting by mismatch-specific endonuclease represented the presence of compound heterozygous variations (c.-78 A>G with c.2 T>A) (Fig. 2H). Similarly, the 193 bp, 144 bp, 236 bp, and 101 bp due to c.9 C>T polymorphism combined with the c.52 A>T variation (Fig. 2I). The other DNA substitutions were identified by following this principle to predict the locations of the mutation sites. This technique could be the gold standard for the identification of healthy carriers, while for the diagnosis of affected patients direct sequencing probably remains the best approach, also considering the small size of HBB gene.

### Detection of HBB mutations by DHPLC

**Figure 4:**
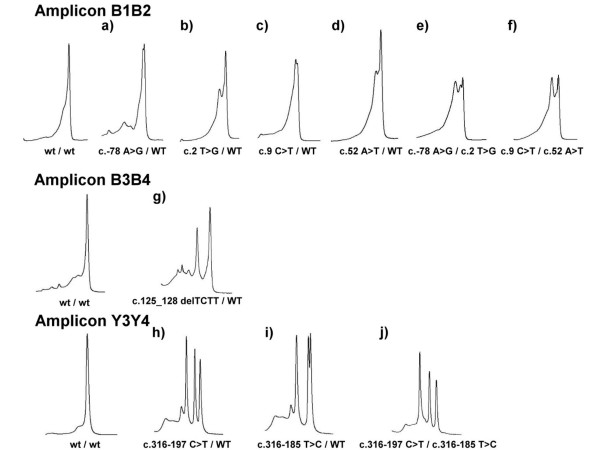
DHPLC chromatography analyses of *HBB *genes for PCR products. (amplicon B1B2 for a, b, c, d, e, f ; B3B4 for g and Y3Y4 for h, i, j). a) an individual with a genotype of c.-78 A>G/WT, b) an individual with a genotype of c.2 T>G/WT, c) an individual with a genotype of c.9 C>T/WT, d) an individual with a genotype of c.52 A>T/WT, e) an individual with a compound heterozygous mutation c.-78 A>G/c.2 T>G, f) an individual with a genotype of c.9 C>T/c.52 A>T, g) an individual with a genotype of c.125_128 delTCTT/WT, h) an individual with a genotype of c.316-197 C>T/WT, i) an individual with a genotype of c.316-185 T>C/WT, and j) an individual with a genotype of c.316-197 C>T/c.316-185 T>C.

DHPLC is a promising tool for mutations detection and gene quantification [34-36]. Previously, we developed a powerful and rapid PCR-DHPLC assay for detection of *HBB *mutations [37]. However, DHPLC had better sensitivity and specificity in the analysis of heteroduplexes in PCR fragments shorter than 500 bp. In an attempt to increase sensitivity, we modified and optimized the PCR primers and DHPLC conditions for this study (Table 2), providing a significant advantage to this technique. The modified assay was used for identification of various genotypes of the *HBB *gene (Figure 4) which were confirmed by direct sequencing.

**Table 2:** Primers used for DHPLC and mismatch-specific endonuclease cleavage analysis of the *HBB *gene

***Primer Name***	***Sequence (5'-3')***	***PCR Length (bp)***	***Anneal Tm (°C)***	***DHPLC Tm (°C)***	***DHPLC %B *** ***Start/end***	***5'-Label***
B1	gacaggtacggctgtcatca	337	56	61	55–64 4.5 min	FAM
B2	gtctccacatgcccagtttc					HEX
B3	gaaactgggcatgtggagac	287	56	61	55–64 4.5 min	FAM
B4	agcttgtcacagtgcagctc					HEX
Y3	gtgtacacatattgaccaaa	423	56	56	56–65 4.5 min	FAM
Y4	agcacacagaccagcacgt					HEX

*HBB *genes could contain one or more mutations that could affect the results of DHPLC. This results in complex chromatography as shown in Figure 4, and failure to easily determine the genotype.

## Discussion

Heteroduplex analysis based on DHPLC and mismatch-specific endonuclease with capillary electrophoresis both have the ability to detect unknown mutations. At present, direct sequencing is the gold standard and its use with multi-capillary electrophoresis, allows for high-throughput automation. Direct sequencing analysis is almost 100% sensitive for the detection of point mutations, small insertions, and deletions. The greatest advantage of DHPLC is its high sensitivity. This method shows small changes in peak chromatography according to differences in hydrophoretic mobility between heteroduplex and homoduplex DNA with high reproducibility. The main advantage of the mismatch-specific endonuclease cleavage method is that it can determine the locations of mutations with good sensitivity and without the use of toxic chemicals. Nonetheless, because it uses fluorescence technology, it is more expensive to run. In the present study, we were able to unambiguously identify all of the genotypes in *HBB *variants by either of these methods with 100% sensitivity and specificity.

DHPLC, direct sequencing, and mismatch-specific endonuclease cleavage all require PCR amplification prior to screening. Thus, the major time factor is the machine run time. The DHPLC method can efficiently analyze one PCR product within 10 min. While the mismatch-specific endonuclease method reactions used in this study required single runs on a single capillary electrophoresis machine, 96-capillary sequencers are available that can be used for mismatch-specific endonuclease cleavage and direct sequencing. The 96-capillary machine can process more samples per hour than a DHPLC machine [38].

We calculated the costs of the screening at approximately $6 per patient for two different exons using DHPLC analysis and another $14 for direct sequencing. The cost of the mismatch-specific endonuclease e cleavage mutation detection was calculated at about $9 per exon, thus it cost approximately $18 per patient for two exons.

In the case where a mutation and polymorphism occur in the same PCR amplicon, the variable polymorphism may affect the DHPLC profile and running conditions (Fig. 4); however, the two mutations could be determined by mismatch-specific endonuclease because the mismatch-specific endonuclease cleavage method can accurately identify the base variant and its location (Fig. 2). Nonetheless, the mismatch-specific endonuclease cleavage method is more time consuming and complicated because it requires FAM- and HEX-labeled primers. DHPLC is very simple and rapid and uses unlabeled primers. Data interpretations are easy with both the mismatch-specific endonuclease method and DHPLC. These two methodologies cannot detect large deletions or duplications in genotypes, however, unless multiplex PCR is designed for DHPLC or capillary electrophoresis [39-41]. On the other hand, Multiplex Ligation-dependent Probe Amplification (MLPA) [42] is more generally used to detect large deletions causing beta-thalassemia [43].

## Conclusion

The mismatch-specific endonuclease method for beta-thalassemia mutations detection is merely as a model for other (large) disease genes for which large scale, high-throughput mutation scanning. This study demonstrated that the mismatch-specific endonuclease method was not only a comparatively faster procedure but was also significantly superior to DHPLC as a tool for gene mutation identification. The method is feasible for use in high-throughput systems due to an automated injection. This method is thus well suited for screening and may also reduce costs.

## Methods

### DNA Extraction

DNA samples were obtained from a total of 50 subjects, including 20 carriers, 20 patients, and 10 normal individuals at National Taiwan University Hospital. Genomic DNA was collected from peripheral whole blood using the Chemagic DNA Blood Kit (Chemagen) according to the manufacturer's instructions. The different genotypes of the DNA samples used in the mismatch-specific endonuclease study are shown in Table 1.

### Polymerase Chain Reaction

PCR primers and conditions for each exon to amplify the *HBB *gene were modified according to previously reported methods [37] and are described in Table 2. The PCR techniques for the provided DNA fragments were performed in a total volume of 25 μl containing 50 ng of genomic DNA; 0.12 μM of each primer; 100 μM dNTPs; 0.5 units of AmpliTaq Gold enzyme (Applied Biosystems); 2.5 μl of GeneAmp 10 × buffer II (10 mmol/L Tris-HCl, pH 8.3, 50 mmol/L KCl), in 2 mmol/L MgCl2 as provided by the manufacturer. Amplification was performed in a multiblock system (MBS) thermocycler (ThermoHybaid). PCR amplification began with denaturation at 95°C for 5 min, followed by 35 cycles of denaturation at 94°C for 30 s, annealing at 56°C for 30 s, extension at72°C for 45 s, and a final extension step at 72°C for 10 min.

### DHPLC Analysis

Mutations screening was performed using the Transgenomic Wave Nucleic Acid Fragment Analysis System (Transgenomic Inc.) with a C_18 _reversed-phase column, which was based on 2-μm nonporous poly (styrene/divinylbenzene) particles (DNASep column, Transgenomic Inc.). PCR products were analyzed in a linear acetonitrile gradient with triethylammonium acetate as the mobile phase, using buffer A (0.1 M TEAA) and buffer B (0.1 M TEAA with 25% acetonitrile) (WAVE Optimized, Transgenomic Inc.). Heteroduplex analyses were performed according to the manufacturer's protocol and previous studies [37,44,45].

### Purification of PCR Products

In the mismatch-specific endonuclease process, the Microcon YM-100 system (Millipore Corp.) was used to remove excess primers and unincorporated dNTPs to purify the PCR products.

### Mismatch-specific Endonuclease Method

After PCR amplification and purification, the PCR product was digested by mismatch-specific endonuclease using the SURVEYOR kit with the fluorescent capillary electrophoresis system (Transgenomic). The PCR products were assayed in a total volume of 60 μl containing 15 μl of PCR product, 6 μl of 10 × Surveyor Nuclease reaction buffer, 1 μl of Surveyor Nuclease Enhancer W, and 1 μl of Surveyor Nuclease W (Surveyor) as provided by the manufacturer. Mismatch-specific endonuclease digestion was performed at 42°C for 20 min, followed by adding 6 μl of stop solution as provided by the manufacturer.

### Capillary Electrophoresis

The final mismatch-specific endonuclease digestion product (2 μl) was diluted with 10 μl of Hi-Di formamide (Applied Biosystems) and 0.25 μl of ROX size standard (Applied Biosystems). Samples were heated at 95°C for 5 min and cooled on ice for 5 min. Each mixture was injected into the ABI PRISM 310 Genetic Analyzer (Applied Biosystems) and was separated across a capillary containing POP-4 polymer (Applied Biosystems). The results were analyzed using GeneScan application software (Applied Biosystems) according to the manufacturer's protocol [46].

### Sequencing

For sequencing, the PCR products were purified by solid-phase extraction and bi-directionally sequenced with the Applied Biosystems Taq DyeDeoxy terminator cycle sequencing kit (Applied Biosystems) according to the manufacturer's instructions. Sequencing reactions were separated on a PE Biosystems 373A/3100 sequencer.

## Authors' contributions

C–CH and Y–NS performed the molecular genetics studies and drafted the manuscript. C–YL, Y–HC and C–HC participated in the molecular genetics studies. W–FC and C–AC performed the clinical characterization of the patients. W–LL participated in designing the study. C–NL conceived of the study, participated in its design and coordination, and helped draft the manuscript. All authors read and approved the final manuscript.
